# Behavior Responses to Chemical and Optogenetic Stimuli in *Drosophila* Larvae

**DOI:** 10.3389/fnbeh.2018.00324

**Published:** 2018-12-21

**Authors:** David A. Clark, Seth R. Odell, Joanna M. Armstrong, Mariah Turcotte, Donovan Kohler, America Mathis, Deena R. Schmidt, Dennis Mathew

**Affiliations:** ^1^Department of Biology, University of Nevada, Reno, NV, United States; ^2^Integrated Neuroscience Graduate Program, University of Nevada, Reno, NV, United States; ^3^Department of Mathematics & Statistics, University of Nevada, Reno, NV, United States

**Keywords:** *Drosophila* larva, olfaction, olfactory receptor neuron, optogenetics, behavior

## Abstract

An animal’s ability to navigate an olfactory environment is critically dependent on the activities of its first-order olfactory receptor neurons (ORNs). While considerable research has focused on ORN responses to odorants, the mechanisms by which olfactory information is encoded in the activities of ORNs and translated into navigational behavior remain poorly understood. We sought to determine the contributions of most *Drosophila melanogaster* larval ORNs to navigational behavior. Using odorants to activate ORNs and a larval tracking assay to measure the corresponding behavioral response, we observed that larval ORN activators cluster into four groups based on the behavior responses elicited from larvae. This is significant because it provides new insights into the functional relationship between ORN activity and behavioral response. Subsequent optogenetic analyses of a subset of ORNs revealed previously undescribed properties of larval ORNs. Furthermore, our results indicated that different temporal patterns of ORN activation elicit different behavioral outputs: some ORNs respond to stimulus increments while others respond to stimulus decrements. These results suggest that the ability of ORNs to encode temporal patterns of stimulation increases the coding capacity of the olfactory circuit. Moreover, the ability of ORNs to sense stimulus increments and decrements facilitates instantaneous evaluations of concentration changes in the environment. Together, these ORN properties enable larvae to efficiently navigate a complex olfactory environment. Ultimately, knowledge of how ORN activity patterns and their weighted contributions influence odor coding may eventually reveal how peripheral information is organized and transmitted to subsequent layers of a neural circuit.

## Introduction

Animals navigate complex olfactory environments in search of food and mates. While tracking odors in natural environments, the olfactory system must account for not only odor identity but also increases and decreases in odor concentrations that are characteristic of turbulent plumes (Zimmer-Faust et al., 1995; Vickers, 2000) and for navigating concentration gradients in non-turbulent situations (Gomez-Marin et al., 2011). The olfactory system must further account for the different temporal profiles of stimuli that result from the physicochemical properties of the odorant and receptivity characteristics of the sensory membrane (Kaissling, 2013; Martelli et al., 2013; Grillet et al., 2016; Larter et al., 2016). Olfactory information in the environment is first sensed by odor receptors expressed in the dendrites of first-order olfactory receptor neurons (ORNs; Buck and Axel, 1991; Clyne et al., 1999). The chemical interaction between the odor receptor and odorants is converted into electrical signals (Buck and Axel, 1991; Couto et al., 2005; Fishilevich and Vosshall, 2005; Kreher et al., 2005, 2008; Sato et al., 2008; Smart et al., 2008; Wicher et al., 2008; Yao and Carlson, 2010; Manzini and Korsching, 2011; Dalton and Lomvardas, 2015). These electrical signals are encoded at various levels of the olfactory circuit, eventually eliciting a behavioral response (Wilson et al., 2004; Fishilevich et al., 2005; Chalasani et al., 2007; Gomez-Marin et al., 2011; Turner et al., 2011; Gomez-Marin and Louis, 2014). Despite the accumulating evidence regarding ORN responses to odorants across species, relatively little is known regarding ORN responses in relation to the temporal aspects of odor stimuli or the mechanisms by which sensory information is encoded by activity in ORN clusters.

In *Drosophila* larvae, olfactory information is sensed by a small panel of 21 ORNs (Couto et al., 2005; Kreher et al., 2005; Ramaekers et al., 2005; Mathew et al., 2013; Dweck et al., 2018). These 21 ORNs send projections into the larval antennal lobe (LAL)—an olfactory neuropil similar to the vertebrate olfactory bulb—where they make connections with 21 uniglomerular projection neurons (PNs), 14 multiglomerular PNs, 14 GABAergic and cholinergic local neurons (LNs), four neuromodulatory neurons, six subesophageal zone (SEZ) neurons, and one descending neuron (Masuda-Nakagawa et al., 2005, 2009, 2010; Ramaekers et al., 2005; Berck et al., 2016). Subsequent processing of information in higher olfactory centers regulates the olfactory behavioral responses of the larva. Recent studies in *Drosophila* (Mathew et al., 2013; Hernandez-Nunez et al., 2015; Newquist et al., 2016) and mammalian systems (reviewed in Yagi, 2013) have suggested that ORNs exhibit functional diversity, in that individual ORNs, upon activation, elicit different compositions of behavioral responses. While some evidence indicates that a subset of sensory neurons is sufficient for functional output (Kreher et al., 2008), we wondered whether the 21 larval ORNs contribute to olfactory behavior in 21 different ways, or whether their contributions can be grouped into a small number of subsets. Such knowledge is critical for both theoretical and applied approaches to understanding olfactory information processing within olfactory circuits.

In the present study, we aimed to determine whether larval ORNs can be grouped into subsets based on their impact on larval navigation. Based on the findings of recent studies (Kreher et al., 2008; Ince et al., 2013), we hypothesized that larval ORNs can be grouped into a small number of functional subsets. Aspects of this study were made possible by the recent development of a technique that enables the researcher to track larval navigation while simultaneously activating individual olfactory neurons using optogenetics (Klapoetke et al., 2014; Hernandez-Nunez et al., 2015; Clark et al., 2018). Optogenetic activation with *CsChrimson* allows for specific and reliable activation of individual ORNs, and also allows us to vary the temporal patterns of their activation. In this study, chemical (Mathew et al., 2013) or optogenetic methods were used to activate ORNs. A larval tracking paradigm (Gershow et al., 2012; Mathew et al., 2013) was used to quantify larval behavior. Results were evaluated to determine the answers to the following questions: Do larval ORNs distribute into a small number of functional subsets? What properties differentiate ORNs in each functional group? Knowledge of ORN activity patterns and their weighted contributions to the circuit will ultimately reveal how peripheral information is organized and transmitted to subsequent layers of the circuit. Thus, to understand the transformation of olfactory input into behavioral output, a necessary first step is to understand the mechanisms by which sensory information is encoded by the activity of the 21 larval ORNs.

## Materials and Methods

### *Drosophila* Stocks

A Canton-S (CS) line was used as the wild-type line in behavioral experiments. Females from a *UAS-IVS-CsChrimson* (*Drosophila* Stock Center, Bloomington, IN, USA) were crossed to males from an *OrX-Gal4* (where *X* = 7a/42a/42b/45a/45b/47a/67b). After mating and egg laying for 48 h, adults are transferred out. To the surface of the food vial, 400 μL of a mixture containing 400 μM all-trans-retinal (ATR) dissolved in dimethyl sulfoxide (DMSO) and 89 mM sucrose dissolved in distilled water was added and F1 progeny larvae were allowed to grow in the dark. ATR is required to upregulate *CsChrimson* expression. Third-instar larvae (96 h after egg laying) are used for the optogenetics experiments.

### Odorants and Other Reagents

Odorants used in these studies were obtained at the highest purity available (≥98% purity, Sigma-Aldrich Inc. St. Louis, MO, USA). They were diluted in paraffin oil (Sigma-Aldrich Inc. St. Louis, MO, USA) for our studies. High purity Agarose (Apex Bioresearch product purchased from Genesee Scientific Inc.) gel was used to prepare the crawling surface for larvae during chemotaxis behavior experiments. ATR (≥98% purity) was purchased from Sigma-Aldrich Inc. 60 mm filter discs (GE-Whatman) used in the behavior assays were purchased from VWR Inc.

### Behavioral Assays

#### Preparation of Larvae for Behavior Assays

Third-instar larvae (~96 h AEL) are extracted from food using a high-density (15%) sucrose (Sigma Aldrich Inc.) solution. Larvae that float to the surface of the sucrose solution are separated into a 1,000 ml glass beaker and washed four times with distilled water. Washed larvae are allowed to rest for 10 min before subjecting them to behavior assays. The temperature of the behavior room is maintained between 22°C and 23°C and between 45% and 50% relative humidity.

#### Odorant Pre-exposure Prior to Larval Tracking

**Figure 1:**
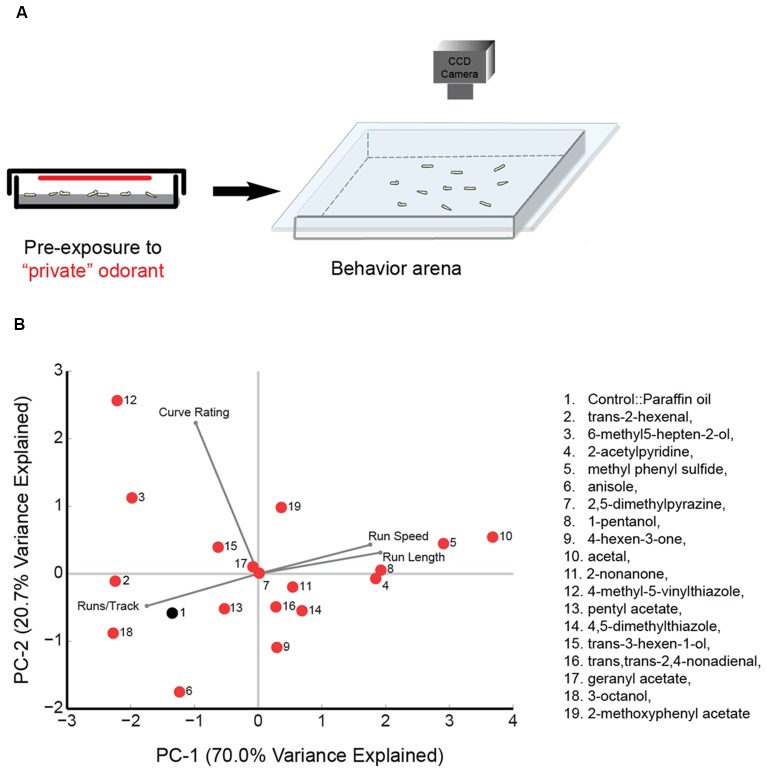
Principal component (PC) analysis of wild type behavior in response to a panel of “best” odorants. **(A)** Cartoon depicting the behavioral paradigm. Approximately 20 larvae are exposed to a “best” odorant in a 6 cm agarose petri-dish. The odorant is placed on a 60 mm Whatman filter paper attached to the ceiling of the plate. After pre-exposure, larvae are transferred to the center of a 22-cm × 22-cm square agarose petri-dish. The chamber is sealed by placing a clear glass plate over the arena. Movement of larvae is recorded with a CCD camera. **(B)** The 18 olfactory receptor neuron (ORN) activators (red circles) and paraffin oil (black circle) are mapped in a behavior space (biplot). *Canton S* (wild-type) larvae were tested against each odorant. Shown are the first two PCs of a multi-dimensional behavior space made up of four navigational descriptors measured at 10^−2^ concentration of odorants (no. of runs per trajectory, run length, run speed, and curve rating). Each descriptor was converted to its Z-score for the full data set. The vectors in the biplot represent the coefficients of the four descriptors (variables) on the PCs. Variances explained by the two PCs are listed. PC1 and PC2 are 70.0% and 20.7%, respectively.

Odorant pre-exposure prior to larval tracking was conducted by placing approximately 20–25 larvae in a 60 mm petri dish along with a small piece of wet Kim wipe. Test odorant [50 μL, 10^−2^ (vol:vol)] was spread uniformly on a 60 mm Whatman filter paper, which was then placed on the inside part of the petri-dish lid. For each trial, approximately 20 larvae were exposed to the odorant for 30 s before being transferred to an empty tracking arena (22-cm × 22-cm) and allowed to roam freely in the absence of any odorant cue (The technique is summarized in Figure 1A). The pre-exposure experiment for each odorant was repeated for 10 trials (with a different set of approximately 20 larvae for each trial) and between 80 and 120 larval tracks for each experiment were analyzed. The order of odorant pre-exposure experiments was randomized.

#### Larval Tracking Assay

Larval tracking assay was conducted as described previously in Mathew et al. (2013). Briefly, approximately 20 third-instar larvae were placed in the center of a 22-cm × 22-cm square petri dish layered with 1.5% agarose. Larval movements were monitored within the experimental arena under dark-field illumination with infra-red LEDs (850 nm, outside the range of larval phototaxis. Environmental lights Inc. San Diego, CA, USA). Images were recorded at 2.3 frames per second using a Monochrome USB 3.0 camera (Basler Ace series, JH Technologies, San Jose, CA, USA) fitted with an IR long-pass 750-nm filter and an 8-mm F1.4 C-mount lens (JH Technologies, San Jose, CA, USA; Figures 1A, 3A). Each pixel in the captured image corresponded to a 0.119-mm square of the experimental arena.

**Figure 2:**
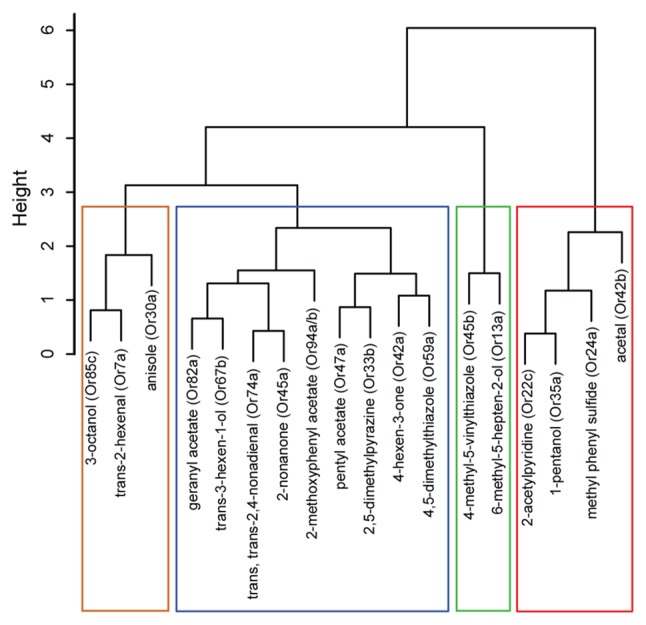
Hierarchical cluster analysis of ORNs based on their functional contributions. Dendrogram depicting a hierarchical cluster analysis performed on navigational data collected from the tracking assay in Figure 1. The vertical axis gives the distance between clusters when they were merged.

**Figure 3:**
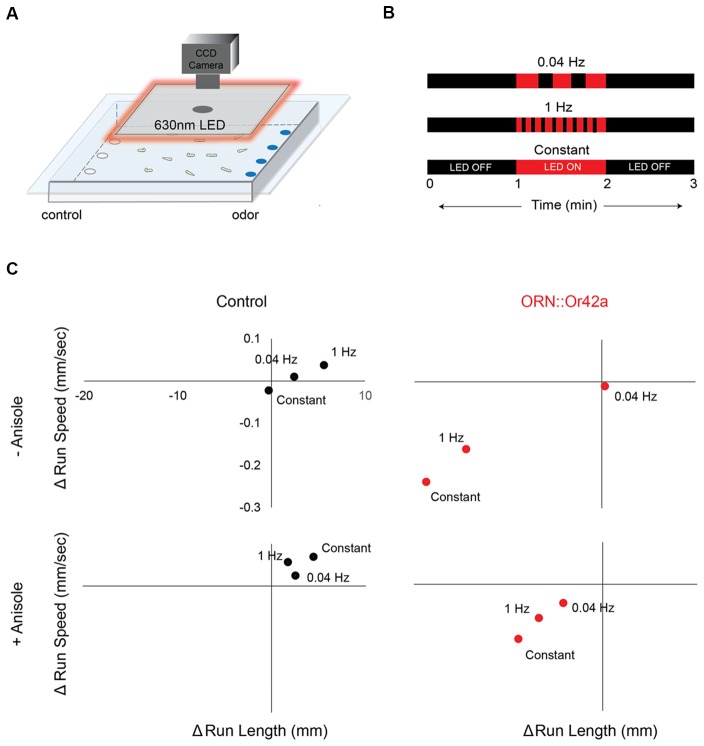
Different temporal patterns of ORN activation elicits different behavioral responses.** (A)** Cartoon depicting the behavioral paradigm. Approximately 20 third-instar larvae expressing *CsChrimson* in a single pair of ORNs are placed in the center of a 22-cm × 22-cm agarose petri-dish. Their movement towards or away from an odor source is recorded with a CCD camera. A square panel of 630 nm LEDs fitted around the CCD camera is used to stimulate *CsChrimson* expressing ORNs in the larvae. **(B)** Three different 630 nm light stimulus protocols are shown. Each 3 min protocol includes LED OFF for the first minute, LED ON for the second minute and LED OFF for the third minute. Each protocol involves a different frequency of light stimulus during the LED ON period (0.04 Hz or 1 Hz or Constant). **(C)** The change in average run length (*x*-axis) is plotted against the change in average run speed (*y*-axis) between min 2 (LED ON) and min 3 (LED OFF). Behavior response of control larvae (left panel; black circles) and larvae expressing *CsChrimson* in ORN::42a (right panel; red circles) to the three different protocols of light stimuli are shown. Top panel shows behavior responses in the absence of anisole. Bottom panel shows behavior responses in the presence of anisole (10^−3^ vol:vol). Significant difference in behavior response was observed in case of test larvae subjected to the different stimulation protocols (*n* = 8; *p* < 0.05, Students *t*-test).

#### Optogenetics Assay

The optogenetics assay was conducted in a (35”L × 24”W × 26”H) behavior arena. A square panel of red LED light strips connected in series and fed into an optocoupler relay (SainSmart 16-Channel Relay Module) is mounted around the CCD camera and suspended from the ceiling of the behavior arena (Figure 3A). The LED strips are controlled by a Raspberry Pi microprocessor. A Linux based operating system (Ubuntu Mate) was installed and configured on the Raspberry Pi microprocessor before connecting the optocoupler relay to the LED strips. A Rigol DP832 power supply provides power for the LED strips and optocoupler. To ensure homogenous irradiance, we measure absolute irradiance at the surface of the arena using a Jaz spectrometer (Ocean Optics) and determined it to be ~1.3 W/m^2^ throughout the surface of the arena.

In order to express *CsChrimson* in a single pair of ORNs, virgin females from a *UAS-IVS-CsChrimson* line are crossed to males from an *OrX-Gal4* line (“X” corresponds to one of 21 larval Or genes that is uniquely expressed in each of 21 pairs of ORNs). *UAS-IVS-CsChrimson* larvae are used as control animals in these experiments. Once male and female flies in a cross are allowed to mate and lay eggs for 48 h, adults are transferred to a fresh vial. An aliquot (400 μL) of 40 mM ATR is then added to the food vial containing the eggs. ATR is a cofactor required for upregulating of CsChrimson expression. Once ATR is added to the food vials containing eggs, the vials are incubated in the dark for an additional 72 h. Third-instar larvae (~120 h after egg laying) are extracted from the food using a high density (15%) sucrose solution, washed three times in distilled water, and allowed to rest for 10 min before starting the assay. To conduct the optogenetics assay, approximately 20 third-instar larvae expressing *CsChrimson* in a single pair of ORNs are placed in the center of a 22-cm × 22-cm square petri dish layered with 1.5% agarose. *CsChrimson* expressing larval ORNs are activated by shining red LED light (630 nm, 1.3 W/m^2^ intensity).

Each experiment lasts for 3 min. During the first minute, there is no light stimulus and larvae are crawling in a dark arena. During the second minute, larvae are exposed to a red light stimulus. Finally, in the third minute, the light stimulus is turned off. Using the Ubuntu mate operating system, we programmed three temporal variations of red LED light stimulus during the second minute: 0.04 Hz, 1 Hz, and constant (Figure 3B). Detailed descriptions of how to construct the behavior arena and the LED platform, and how to process the data for the optogenetics assay are provided in Clark et al. (2018).

### Data Processing and Statistical Analysis

#### Navigational Parameters

For analyzing larval navigation in the tracking assay, positions of larvae for the entire duration of the assay or for each minute of the assay (in the case of optogenetics experiments) were extracted from video recordings and larval “trajectories” were reconstructed by using custom routines written in MATLAB (Mathworks Inc., Natick, MA, USA, RRID:SCR_001622). Between 80 and 120 trajectories were analyzed for each trial. The response index (*<v_x_>/<s>*) was defined as the mean velocity of the larva in the *x*-direction (*<v_x_>*) divided by the mean crawling speed (*<s>*) as described previously in Gershow et al. (2012). Based on some navigational statistics such as speed, path curvature, heading angle, we segmented trajectories into alternating sequences of runs and turns. Runs were defined as continuous periods of forward movement. Turns separated successive runs. Turns were flagged when the change of trajectory orientation angle was >45°. Further statistics were applied to individual runs to calculate run direction (average orientation of runs in a scale of 0 to ±180, with “0” → towards the odor and ±180 → away from the odor), run length, and run speed. Run length and run speed were further calculated for runs (toward) odor (all runs that orientated between +45° and −45°) and for runs (away) from odor (all runs that oriented between +135° and −135°). Run ratio was calculated as the mean run length of runs toward odor divided by the mean run length of runs away from odor. Curvature was defined as the average change of direction of each track (Curvature of 0 indicates no net change in direction). Curve rating was defined as the total length of a trajectory divided by its total displacement (curve rating of 1 indicates a perfectly linear track).

#### Cluster Analysis

A hierarchical cluster analysis was performed using R version 3.3.1 (R Core Team, 2016) on the following navigational data collected from the tracking assay: no. runs per trajectory, run length, run speed, and curve rating (parameters defined in the previous section). Specifically, hclust function within the stats package in R was used. The default settings (complete linkage method and Euclidean distance to measure the distance between clusters) on the mean data for each of the 18 ORNs were used. To normalize the data and allow for comparisons across parameters, each of the four sets of navigational data was centered by its mean and then divided by its standard deviation. Results were visualized as a dendrogram by successively joining each pair of clusters in the order they were merged in the clustering algorithm. The vertical axis gives the distance between clusters when they were merged. Robustness of the clustering was tested by assigning random noise to the data using the “jitter” function in Matlab and repeating the analysis 100 times.

#### Statistics

Statistical analyses were performed using Statistica (Statsoft Inc., Tulsa, OK, USA RRID: SCR_014213). All data were expressed as mean ± standard error of the mean. The Kolmogorov-Smirnoff test was used to assess the normality of distribution of the investigated parameters. Parameters derived from larval tracks did not follow a normal distribution when categorized either by ORN type, time (minutes), presence or absence of a directional cue, or stimulation frequency. Intergroup analysis was done with the Kruskal-Wallis multiple comparison test. Differences were considered significant at *P* < 0.05.

## Results

### Pre-exposure to ORN Activators Elicits Different Compositions of Larval Behavior

*Drosophila* larval navigation is composed of discrete behavioral elements such as head sweeps, runs, and turns (Luo et al., 2010; Gomez-Marin et al., 2011; Gershow et al., 2012). Recent studies have argued that individual larval ORNs are functionally diverse, providing different contributions to larval navigation (Mathew et al., 2013; Hernandez-Nunez et al., 2015; Newquist et al., 2016). In the present study, we aimed to determine whether larval ORN activators could be classified based on their functional contributions. We first measured the impact of activating ORNs on larval behavior. To activate ORN pairs, we utilized a panel of 18 previously published odorants (Mathew et al., 2013). In an electrophysiology assay, each of the 18 odorants elicited a strong, specific physiological response from individual larval odor receptor (Or) expressed in the adult empty neuron system (Or7a::trans-2-hexenal; Or13a::6-methyl5-hepten-2-ol; Or22c::2-acetylpyridine; Or24a::methyl phenyl sulfide; Or30a::anisole; Or33b::2,5-dimethylpyrazine; Or35a::1-pentanol; Or42a::4-hexen-3-one; Or42b::acetal; Or45a::2-nonanone; Or45b::4-methyl-5-vinylthiazole; Or47a::pentyl acetate; Or59a::4,5-dimethylthiazole; Or67b::trans-3-hexen-1-ol; Or74a::trans, trans-2,4-nonadienal; Or82a::geranyl acetate; Or85c::3-octanol; Or94a,b::2-methoxyphenyl acetate). The Ors targeted in our study include most of the Ors previously shown to be functional during the larval stage of *Drosophila melanogaster* (Couto et al., 2005; Kreher et al., 2005, 2008). Since this panel of 18 “best” odorants have different volatilities and thus give rise to odor gradients of different strengths, we chose not to use the traditional two-choice assay format as used in Mathew et al. (2013) to measure the impact of ORN activation on behavior. Instead, we activated ORNs by pre-exposing wild-type larvae to each of these odorants and measured the resulting behavioral responses (Figure 1A). We note that while each odorant, when tested at a concentration of 10^−4^ vol:vol, elicited a specific response from a single larval odor receptor in the electrophysiology assay, it is possible that they may activate multiple ORNs when exposed at closer proximity and for longer duration in the behavior assay. We will temper the interpretations of our results accordingly. The pre-exposure experiment for each odorant was repeated for 10 trials and approximately 80–120 trajectories were analyzed for each experiment.

We investigated the impact of activating ORNs on navigational parameters such as the number of runs per trajectory, run length, run speed, and curve rating. Since there was no directionality in the behavioral arena due to lack of an odor source, we did not calculate a response index. However, run length and run speed are positively correlated with attractive response toward an odor source while number of runs per trajectory is negatively correlated with attractive responses (Gershow et al., 2012; Mathew et al., 2013; Gomez-Marin and Louis, 2014). All data for this experiment is provided in Supplementary Table S1. A four-dimensional behavior space was used to map the behavior elicited by each of the 18 odorants and paraffin oil control, according to the four navigational parameters. We then examined the position of odorants in this behavior space. The first two principal components (PCs) are shown in Figure 1B. Also shown in the biplot are vectors that represent the coefficients of the four behavioral descriptors (variables) on the PCs. For example, acetal (odor #10) elicits long run lengths and high run speed in larvae and fewer number of runs per track. On the other hand, trans-2-hexenal elicits shorter run lengths and low run speed in larvae and a greater number of runs per track (Figure 1B, Supplementary Table S1). The average Euclidean distance between all odorants was 2.56 ± 0.09 a.u. However, some odorants were mapped relatively far from one another. For instance, 4-methyl-5-vinylthiazole (activates Or45b) mapped furthest from acetal (activates Or42b), with a Euclidean distance of 6.10 a.u. between the two odorants. We also noted that some odorants grouped close together in this space. For instance, 2-nonanone (activates Or45a); trans, trans-2,4-nonadienal (activates Or74a); and 4,5-dimethylthiazole (activates Or59a) were mapped very close to one another: the average Euclidean distance among the three odors was only 1.40 ± 0.48 a.u. Similarly, the Euclidean distance between 2-acetylpyridine (activates Or22c) and 1-pentanol (activates Or35a; 0.37 a.u.) was lower than the average distance between all odorants. These findings suggest that some odorants in the selected panel elicit similar patterns of behavior while some elicit very different patterns of behavior.

We then examined whether the position of odorants in the PCA map (Figure 1B) could be used, as a first pass, to predict potential relationships among the ORN activators. Thus, we conducted a hierarchical cluster analysis on the navigational data from the tracking assay for each of the 18 odorants. (Figure 2). Typically, in such analyses, the reliability of clustering is proportional to branch heights in the dendrogram. Thus, we focused on the longer branches (those with heights greater than four units) and depending on the branch-height cutoff used, we observed two to four distinct clusters in the data. For the rest of the study, we chose to focus on the grouping with four clusters. To further test the robustness of this clustering structure, we used the “jitter” function in R version 3.3.1 (R Core Team, 2016) to add random noise to the behavioral data and repeated the analysis 100 times. In every case, the components of the four clusters remained the same and we found only slight differences in arrangements of ORNs within each cluster due to their short branch lengths. With the caveat in mind that individual odorants could activate multiple ORNs, from this initial analysis, we estimated that the best odorants could be classified into four groups based on the behavioral responses that they elicit from wild-type larvae.

### ORNs Express Temporal Diversity

To determine the role of individual clusters of larval ORNs during navigational behavior (Figure 2), we examined the behavioral impact of activating individual ORNs that correspond to activators from each cluster. While low concentrations of each odorant elicit a physiological response from its cognate ORN only, higher concentrations of each odorant—which are typically utilized for conventional behavior assays—elicit physiological responses from multiple ORNs (Hallem and Carlson, 2006; Kreher et al., 2008; Mathew et al., 2013). Furthermore, odorants exhibit various levels of volatility, complicating interpretation of behavioral studies, which depends on the formation of stable odor gradients (Monte et al., 1989; Gershow et al., 2012). To activate individual ORNs, we utilized a recently reported optogenetic technique (Hernandez-Nunez et al., 2015; Clark et al., 2018). The optogenetic approach is also advantageous in that one can alter the frequency of light stimulation, enabling us to vary the temporal patterns of ORN activation, which is difficult to achieve using odor stimuli. In a recent study, Hernandez-Nunez et al. (2015) demonstrated a direct correspondence between ON/OFF pulses of *CsChrimson* activation and the physiological activity (spiking) induced in larval ORN expressing *CsChrimson*. They found that optogenetic activation of two separate ORNs (expressing Or42a and Or45a) reliably and reproducibly induced spike trains during exposure to three different temporal pulses (0.2, 0.5, and 1 s) of red light.

We investigated the roles of seven ORNs, each expressing one of the following receptors: Or42b from cluster 1; Or45b from cluster 2; Or7a from cluster 3; and Or42a, Or45a, Or47a, and Or67b from cluster 4 (Figure 2). In the pre-exposure assay, the odorant soaked filter paper covered the ceiling of the assay plate, causing the odor to spread uniformly across the arena. Thus, this behavioral assay was performed in the absence of any directional cue. However, directional cues are advantageous in that they provide a choice point for the larvae, thereby increasing the dimensionality of behavioral measures (i.e., towards or away from odor). Thus, directional cues were provided for all subsequent behavioral assays. In these assays, anisole (10^−3^ vol:vol) was applied to one side of the assay plate. We chose anisole for two reasons: (1) in previous electrophysiology studies, anisole was shown to activate few ORNs (Hoare et al., 2011; Mathew et al., 2013); and (2) when tested in a behavior assay, anisole elicited changes in larval navigational parameters but generated an overall weak response index (statistically insignificant relative to a response index of 0; Newquist et al., 2016). We genetically expressed *CsChrimson* in individual pairs of ORNs (using individual Or-Gal4s). ORNs expressing *CsChrimson* were activated by shining red LEDs (630 nm, 1.3 W/m^2^ intensity) on larvae navigating in the presence of the anisole gradient (Figure 3A; Clark et al., 2018). We varied our stimulus to alternate between lights OFF and ON for a total of 3 min (Figure 3B). Larval movement was tracked in the presence and absence of *CsChrimson* activation and in the presence and absence of the directional cue. We then measured navigational parameters as described in previous studies (Gershow et al., 2012; Mathew et al., 2013; Newquist et al., 2016; Clark et al., 2018).

We observed the largest change in behavioral responses when the stimulus was switched from lights ON (min 2) to lights OFF (min 3) for larvae expressing *CsChrimson* in ORN::42a. We then investigated whether different temporal patterns of ORN activation resulted in different behavioral responses. We programmed three different temporal patterns of light stimulation [slow (0.04 Hz), fast (1 Hz), and constant] during the lights ON period (Figure 3B). In the graphs shown in Figure 3C, we plotted the changes in run length (*x*-axis) and run speed (*y*-axis) as the stimulus changed from ON to OFF. This experiment was carried out both in the absence (top panel in Figure 3C) and presence (bottom panel in Figure 3C) of the directional cue, anisole (10^−3^ vol:vol). No significant differences in behavior were observed in control larvae for any pattern of light stimulation. However, significant differences in behavior were noted between constant and 0.04 Hz stimulation and between constant and 1 Hz stimulation for larvae expressing *CsChrimson* in ORN::42a (*P* < 0.05, Student’s *t*-test). These differences were observed regardless of the presence or absence of the directional cue.

**Figure 4:**
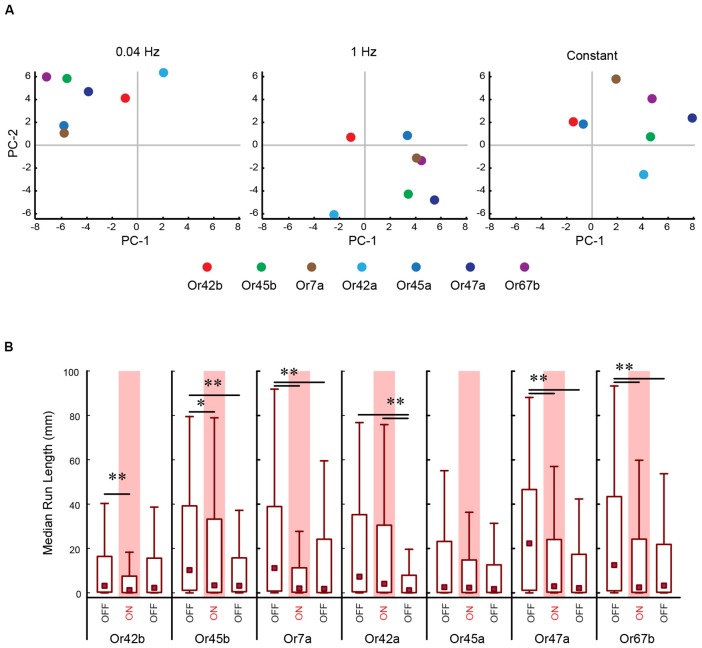
PC analysis of larval behavior responses to different patterns of optogenetic stimulation. **(A)** The seven *CsChrimson* expressing ORNs tested in the optogenetics experiment are mapped in a behavior space. Each color coded circle represents a *CsChrimson* ORN expressing a different larval odor receptor. Each graph is constructed such that the control ORN (not expressing *CsChrimson*) maps to the origin. Shown in each graph are the first two PCs of a multi-dimensional behavior space made up of 10-navigational descriptors (response index, no. runs per trajectory, run length (toward), run length (away), run speed (toward), run speed (away), run ratio, run direction, curvature, and curve rating for each minute of a 3 min assay). Each descriptor was converted to its Z-score for the full data set. Three behavior spaces are plotted. Each graph relates to behavior measured during one of three firing patterns (0.04 Hz, 1 Hz, Constant firing). Variance explained by the first three PCs and the rest of the variances explained by the other eight components for each graph are shown in Table 1. **(B)** The median run length for each minute in the Constant firing pattern was determined and plotted. Boxes are interquartile range. Bars are the non-outlier range as defined by Statistica (Statsoft Inc., Tulsa, OK, USA). *p < 0.001, and **p < 0.0001.

To perform a more comprehensive analysis of the impact of different patterns of light stimulation on individual ORNs, we repeated the above experiment for all seven ORNs. In this analysis, we measured 10 previously described navigational parameters: response index, number of runs per trajectory, run length (toward), run length (away), run speed (toward), run speed (away), run ratio, run direction, curvature, and curve rating for each minute of a 3 min assay for each stimulation frequency (Gershow et al., 2012; Mathew et al., 2013; Newquist et al., 2016). All data for this experiment is provided in Supplementary Table S2. We used PC analysis to construct a multi-dimensional behavioral space based on these 10 navigational parameters for each minute of the experiment (totaling 30 combinations per behavioral space). We mapped each ORN in the constructed space. For ease of visualization, the data from this multi-dimensional analysis was reduced to two dimensions, as shown in Figure 4A. We conducted separate analyses for each pattern of light stimulation (0.04 Hz, 1 Hz, constant; Figure 4A). A visual inspection of these graphs revealed that ORNs were clustered differently under different light stimulation patterns. To more objectively investigate whether different patterns of light stimulation led to different behavioral outputs, we measured the median Euclidean distances between each combination of ORNs in this space. Our analysis revealed that the positions of ORNs in relation to each other in the multi-dimensional spaces were different for each stimulation pattern. For example, the nearest neighbor of Or7a (Figure 4A, yellow dot) at the 0.04 Hz stimulation frequency is Or45a (blue dot; 6.80 a.u.). However, the nearest neighbor of Or7a at the 1 Hz stimulation frequency is Or67b (purple dot; 6.84 a.u.) This relationship between Or7a and Or67b is also observed at the constant stimulation pattern (5.95 a.u.).

**Table 1:** Percentage of variance explained by first few principal components (PCs) in Figure 4A.

Stimulus	PC-1 (%)	PC-2 (%)	PC-3 (%)	All other PCs (%)
0.04 Hz	37.3	20.3	13.8	28.6
1 Hz	28.4	23.7	16.6	31.3
Constant	35.1	21.4	14.0	29.5

Next, we asked whether optogenetic stimulation differently impacted individual ORNs. To do so, we examined one example of a change in larval response (run length) in response to one pattern of light (constant) stimulation (Figure 4B). Five of the seven ORNs tested exhibited a drop in run length from the first minute to the second minute. The most dramatic change was observed in Or7a [min 1: 11.2 mm (0.781–38.9 mm); min 2: 2.07 mm (0.278–11.3 mm); *P* = 1.95 × 10^−7^]. There is a minor increase of run length in the third minute of Or7a which is still significantly less than in min 1 [min 3: 1.86 mm (0.132–24.2 mm), *P* = 3.41 × 10^−5^]. Mapping furthest from Or7a in this behavior space is Or42a (8.89 a.u.) which shows a different pattern in run length during the trial. There is an insignificant drop in run length from min 1 [7.30 mm (0.435–35.2 mm)] to min 2 [4.05 mm (0.212–30.5 mm), *P* = 0.113]. However, there is a significant drop in run length from min 1 and 2 to min 3 [1.27 mm (0.0909–7.93 mm), *P* = 1.76 × 10^−9^; median (interquartile range), Kruskal-Wallis multiple comparisons test]. Together, the data in Figures 3C, 4A strongly suggest that different temporal patterns of ORN activation result in different behavioral outputs and the data in Figure 4B suggests that optogenetic stimulation differently impacts individual ORNs. These results not only support the claim of functional diversity among first-order ORNs (Figures 1, 2; Mathew et al., 2013; Hernandez-Nunez et al., 2015; Newquist et al., 2016) but also imply a previously undescribed temporal dimension to their function.

### Evidence for Increment- and Decrement-Type ORNs

**Figure 5:**
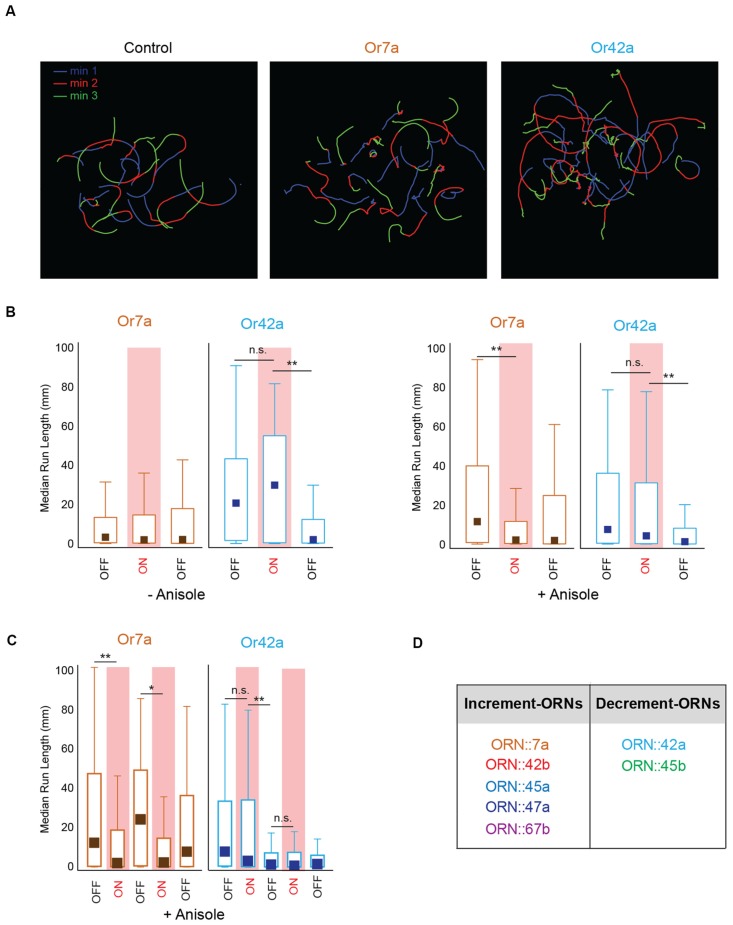
Larval behavior response to stimulus increments and decrements. **(A)** Sample navigational tracks from the 3-min optogenetics assay for control larvae, larvae expressing *CsChrimson* in ORN::7a and in ORN::42a are shown. Each track is color coded to indicate the three stages of the experiment; Blue (min 1, lights OFF), red (min 2, lights ON), and green (min 3, lights OFF). **(B)** Median run length of larvae expressing *CsChrimson* in ORN::7a (brown) or ORN::42a (blue) is measured for every 1-min period during a 3-min assay and plotted on the *y*-axis. Left panel shows behavior responses in the absence of anisole. Right panel shows behavior responses in the presence of anisole (10^−3^ vol:vol). For each experiment, lights are OFF for min 1, ON for min 2 and OFF for min 3. Lights ON periods are shaded in red. **(C)** Median run length of larvae expressing *CsChrimson* in ORN::7a (brown) or ORN::42a (blue) is measured for every 1-min period during a 5-min assay and plotted on the *y*-axis. For each 5-min assay, lights are OFF for the first minute and then alternate between ON and OFF every other minute. A constant light stimulus is used during lights ON period. Boxes (in **B,C**) are interquartile ranges. Bars are the non-outlier range as defined by Statistica (Statsoft Inc., Tulsa, OK, USA). Kruskal-Wallis multiple comparisons test was performed to compare whether the distributions are different from one another. n.s., not significant; *p < 0.01, **p < 0.001. **(D)** Seven larval ORNs classified as Increment or Decrement ORNs based on corresponding larval response to stimulus increments or decrements.

Our optogenetic experiments also suggested that, at least for some ORNs, behavioral outputs were greater when the stimulus changed from ON to OFF than when it changed from OFF to ON. This was also evident when we visually compared individual larval tracks of different genotypes tested in our optogenetic analyses (Figure 5A). Control larvae showed tracks of similar run lengths during min 1 (Lights OFF, Blue), min 2 (Lights ON, Red), and min 3 (Lights OFF, green). However, larvae expressing *CsChrimson* in ORN::7a showed shorter run lengths during min 2 (red tracks) while larvae expressing *CsChrimson* in ORN::42a showed shorter run lengths during min 3 (green tracks). To quantify this observation, we plotted median run lengths for each minute of the 3-min assay for larvae expressing* CsChrimson* in ORN::7a and ORN::42a. Additionally, we compared the behavior in the presence and absence of the directional cue, anisole (10^−3^ vol:vol; Figure 5B). We observed that for both−anisole and +anisole conditions, larvae expressing *CsChrimson* in ORN::42a showed no significant change in median run length when lights changed from OFF [–anisole, min 1: 20.1 mm (1.53–42.3 mm)] to ON [–anisole, min 2: 29.1 mm (1.53–42.3 mm)] (–anisole, min 1 vs. min 2: *P* = 1.00; +anisole, min 1 vs. min 2: *P* = 0.113) but showed a significant drop in run length when light changed from ON to OFF [–anisole, min 3: 1.85 mm (0.246–12.0 mm)] [–anisole, min 2 vs. min 3: *P* = 1.23 × 10^−9^; +anisole, min 2 vs. min 3: *P* = 8.41 × 10^−5^; Figure 5B median (interquartile range), Kruskal-Wallis multiple comparisons test]. On the other hand, larvae expressing *CsChrimson* in ORN::7a showed a significant change in median run length when lights changed from OFF [min 1: 11.16 mm (0.78–38.93 mm)] to ON [min 2: 2.07 mm (0.27–11.29 mm)] (min 1 vs. min 2: *P* = 1.94 × 10^−7^). However, this was observed only in the +anisole condition (Figure 5B, right panel). Next, we wanted to see whether this trend was reproducible over multiple stimulations. We evaluated larval behavior during a 5-min behavioral assay in which the light stimulus was altered each minute (OFF-ON-OFF-ON-OFF). We performed this assay with larvae expressing *CsChrimson* in ORN::7a [an ORN considered to be active in repulsive responses (Kreher et al., 2008)] and ORN::42a [an ORN considered to be active in attractive responses (Kreher et al., 2008; Mathew et al., 2013; Schulze et al., 2015; Boyle et al., 2016)]. We measured the median run lengths of larvae during each minute of lights ON/OFF. The data for this experiment are presented in Figure 5C. Run length significantly decreased when ORN::7a expressing *CsChrimson* was stimulated by a constant pulse of light [min 2: 1.81 mm (0.485–11.2 mm)] relative to pre-stimulation levels [min 1: 11.9 mm (0.404–46.3 mm)] (*P* = 4.10 × 10^−4^), while it reverted back to baseline when the light was turned OFF [min 3: 23.6 mm (0.623–48.0 mm)] (min 1 vs. min 3: *P* = 1.00). This pattern repeated reproducibly in min 4 (lights ON) and min 5 (lights OFF). No significant changes in run length were observed for larvae expressing *CsChrimson* in ORN::42a when the light was turned ON [min 2: 2.93 mm (0.202–33.2 mm)] (min 1 vs. min 2: *P* = 0.753); however, significant decreases relative to pre-stimulation levels [min 1: 7.65 mm (0.466–32.7 mm)] were observed when the light was turned OFF [min 3: 1.23 mm (0.125–7.06 mm)] (*P* = 2.74 × 10^−7^). Run length remained depressed when the light stimulus was altered in subsequent minutes (Figure 5C). These data suggest that ORN::7a drives behavior changes in response to light increments, while ORN::42a drives behavior changes in response to light decrements (we recognize that we only tested constant patterns of stimulation during the lights ON period). We then repeated this experiment for the remaining five ORNs tested above. Based on our results (data not shown), we classified the seven ORNs into two groups: increment-ORNs and decrement-ORNs (Figure 5D).

## Discussion

In the present study, we combined novel methods of ORN activation with high-resolution behavioral analysis to characterize the functional contributions of ORNs in the *Drosophila* larval olfactory circuit. First, we demonstrated that the “best” odorants for larval ORNs can be classified into a small number of groups based on larval behavior responses (Figures 1, 2). Next, we analyzed the properties of individual ORNs within each group. Our findings provide strong evidence to support the conclusions that, at least for some ORNs, different temporal patterns of activation lead to different behavioral outputs (Figures 3, 4), and that some ORNs impact behavior in response to stimulus increments, while others impact behavior in response to stimulus decrements (Figure 5).

### Functional Grouping of ORNs

Recent studies in insects and mammals have suggested that olfactory neurons exhibit functional diversity (Mathew et al., 2013; Yagi, 2013; Hernandez-Nunez et al., 2015; Newquist et al., 2016). We classified 18 “best” odorants for larval ORNs into four groups based on the behavior responses elicited from wild-type larvae. Based on this analysis, we extrapolated that 19 of the 21 larval ORNs could be grouped into four groups based on their contributions to four different behavioral parameters (Figure 2). One caveat of this conclusion is that these results are based on the premise that each odorant used in this study activates individual pairs of larval ORNs. While there is strong electrophysiology evidence to back this premise, we acknowledge that due to the nature of the behavior assay employed, a single odor may activate more than one pair of ORNs. Due to heterogeneity in single-neuron properties, a small but influential subset of neurons is likely sufficient for carrying essentially all information present in the entire population (Ince et al., 2013). A recent study suggested that 81% of behavioral variations in a simple larval two-choice assay could be explained by the activity of only five receptors (Or42a, Or45a, Or74a, Or82a, and Or85c; Kreher et al., 2008). Our findings indicated that four of these five receptors were clustered together (Figure 2). While this raises more questions about the disproportionate influence of one cluster of receptors on larval behavior, our grouping of ORNs based on their function may help to determine how the synaptic weights of sensory neurons are distributed in theoretical models of information coding in neural circuits (Song et al., 2005).

While the molecular or physiological basis for functional differences among larval ORNs remains to be elucidated, recent studies have suggested that functional differences at the neuronal level may arise due to both genetic and non-genetic mechanisms (reviewed in Yagi, 2013). For instance, stochastic expression of homophilic cell adhesion molecules among individual neurons of the same class has been shown to impact the accuracy of axonal projections, synaptic formation, dendritic arborization, and neuronal survival (Esumi et al., 2005; Hirano et al., 2012). Functional diversity may also be generated by the differential expression of critical neurotransmitter receptors at the terminals of sensory neurons. For instance, GABA_B_-receptor levels differ between fruit-fly ORNs tuned to the aversive odorant CO_2_ and those tuned to pheromone detection (Root et al., 2008). One or more of the aforementioned mechanisms likely contribute to the diversity of larval ORNs among individual groups.

### Temporal Patterns of ORN Activation

In natural environments, animals encounter different temporal patterns of odor stimuli, perhaps because different odorants exhibit different adsorption properties at the cuticular surface, causing some odors to linger for longer than others (Martelli et al., 2013). Such differences in the temporal patterns of odor stimuli may also be due to the influence of sensillar events that transport odorant molecules from the surface to ORN dendrites (Kaissling, 2013; Larter et al., 2016). Sniffing rates can also influence odor stimulus dynamics in animals that breathe air. During chemotaxis in *Drosophila* larva, head casting is considered to be an active sampling process analogous to “sniffing” (Gomez-Marin et al., 2011). Most olfactory behavior assays conducted in a lab environment do not take into account this natural temporal variation in odor stimuli. Light-dependent *CsChrimson* activation allowed us to vary the temporal patterns of ORN activation in a controlled manner (Hernandez-Nunez et al., 2015; Clark et al., 2018). We noted that different temporal patterns of ORN activation led to different behavioral outputs (Figures 3, 4). These results support previous studies, which suggest that an animal’s ability to navigate toward an odor is impacted by the temporal pattern in which the odor interacts with its sensory apparatus (Zimmer-Faust et al., 1995; Vickers, 2000; Gomez-Marin et al., 2011).

The olfactory system encodes the average rate and degree of change in odor concentration as well as the ratio of the constituents’ concentrations (Webster and Weissburg, 2001). Insect antennae exhibit a fine-grained temporal resolution capable of tracking small shifts in concentration within an odor plume (Szyszka et al., 2014). The ability of individual ORNs to sense different temporal patterns of stimulation and drive appropriate navigational responses significantly enhances the coding capacity of the olfactory system. This is especially relevant in the case of the larval stage of the fruit-fly, which remains immersed in the food source and continually bathed in food odors. A recent study looked at electrophysiological activity of larval ORNs during short and continuous odor exposures (5 and 20 min). It showed that apart from spike frequency, the temporal information encoded in the activity of ORNs enabled a classifier algorithm (and presumably the animal’s olfactory system) to accurately identify odors. Interestingly, continuous odor exposure (5 and 20 min) did not change the ORN’s ability to encode temporal information (Grillet et al., 2016). Our findings that different temporal patterns of ORN activity elicit different behavioral responses complement existing computational models such as “primacy coding” (Wilson et al., 2017) or “latency encoding” (Brody and Hopfield, 2003), both of which attempt to solve the problem of odor identification via temporal mechanisms. The primacy coding model extends latency encoding models by assuming that only the earliest set of ORNs activated are representative of odors, irrespective of concentration. This model proposes that temporal relationships within a circuit are important only as long as they help to identify these early ORNs. The primacy coding model predicts a basic network architecture that can decode early activity to create a stable representation in deeper regions of the brain. The temporal properties of ORN responses observed in the present study may increase the coding capacity of olfactory networks to determine odor identity. Thus, our results complement other studies that have sought a mechanistic understanding of how animals identify and navigate complex olfactory environments.

### Increment- and Decrement-ORNs

As an animal tracks a turbulent odor plume, it encounters a series of high-concentration pulses interspersed with low- or zero-concentration pulses (Zimmer-Faust et al., 1995; Vickers, 2000). We attempted to simulate this at low levels using optogenetics to turn ORN activation ON and OFF during larval behavior. We noted that some larval ORNs impacted behavior in response to stimulus increments, while others impacted behavior in response to stimulus decrements (Figures 5A–D). While the temporal resolution of this experiment could be further improved, based on the present analysis, we were able to classify seven ORNs as either increment-ORNs or decrement-ORNs (Figure 5D).

We were tempted to refer to these ORNs as ON-neurons and OFF-neurons. However, our characterization of these neurons was based entirely on behavior analyses. Conventionally, ON- and OFF-neurons are defined after careful electrophysiological analyses. True ON-neurons exhibit excitatory responses to stimulus increments while OFF-neurons exhibit excitatory responses to stimulus decrements. Further physiological analyses of individual larval ORNs would be required to confirm our classification of the seven ORNs as ON- or OFF-ORNs (Figure 5D). The concept of ON and OFF sensory neurons has long been established in the mammalian visual system: ON-center ganglion cells respond to light increments, while OFF-center ganglion cells respond to light decrements (Famiglietti and Kolb, 1976). However, only recently has this property been observed for sensory neurons, in insect olfactory systems. Recent studies have indicated that ON- and OFF-ORNs in cockroach antennae provide excitatory responses for increases and decreases in odor concentration respectively (Tichy et al., 2005; Hellwig and Tichy, 2016). Such a mechanism is advantageous for efficient information coding. The antagonism of ON/OFF responses may facilitate instantaneous evaluation of the odor plume to help insects differentiate between concentrations higher and lower than background levels, and to thus fine tune tracking decisions. The cockroach antenna exhibits a bias toward decreasing concentrations, suggesting that the OFF responses are used for accurate tracking (Hellwig and Tichy, 2016). Since *Drosophila* larvae are known to sample their olfactory environments using head casts (analogous to “sniffing”; Gomez-Marin et al., 2011), increment- and decrement-ORNs may play different roles at different points during a sniff cycle. Indeed, the decrement-ORN may aid in resetting the odor identification mechanism at the end of a sniff cycle.

ON and OFF cells have also been described among inhibitory interneurons in the olfactory circuit of adult *Drosophila* (Nagel and Wilson, 2016). The ON and OFF nature of these cells has been attributed to interactions between the synaptic and intrinsic properties of the interneurons. Whether similar diversity exists among larval interneurons—and whether this potential diversity is associated with the diversity of sensory stimuli established in the present study—remain to be determined. The connectivity of the LAL was previously mapped and described by Cardona and colleagues (Berck et al., 2016). For the ORNs tested in the present study, we observed no correlation between the amounts of LN inputs received by ORNs. The median percentage of postsynaptic sites onto LNs from increment- and decrement- ORNs was 2.0% and 2.5%, respectively (Mann-Whitney U, *U* = 95.5, *P* = 0.86). Furthermore, the median percentage of postsynaptic sites onto cognate PNs from LNs is 0.50% for increment- cognate PNs and 4.5% for decrement- cognate PNs (Mann-Whitney U, *U* = 83.0, *P* = 0.47).

### Limitations of the Study

The present study possesses several limitations of note. In the first experiment, we chose a panel of odorants, each of which was shown to specifically activate a different larval Or expressed in an adult expression (empty-neuron) system (Mathew et al., 2013). However, it is likely that, at the concentration and proximity used for larval pre-exposure experiments, the odorants elicit responses from more than one ORN (Hallem and Carlson, 2006; Kreher et al., 2008). There may also be the concern that Ors in larval ORNs might respond differently to odors compared to ORs expressed in the empty neuron system. However, a previous study showed correspondence between electrophysiology responses of Ors expressed in the empty-neuron system and Ors in larval ORNs (Montague et al., 2011). While optogenetics offers a clear solution by enabling precise activation of individual ORNs in a similar manner, the analysis based on functional output in response to natural odor stimuli enabled us to undertake a preliminary classification of larval ORN activators. However, it is less clear whether ORN members of individual clusters instruct different aspects of larval navigation. Our clustering analysis was based on only four behavioral descriptors, as there was no directional cue in the navigation assay. This type of classification may be further improved by considering additional behavioral descriptors based on animal posture (Gershow et al., 2012; Luo et al., 2014). We acknowledge that any study that makes general conclusions regarding the vast olfactory landscape is limited. A recent study established a strong correspondence between short (0.2, 0.5, 1 s) ON/OFF pulses of *CsChrimson* activation and physiological activity (spiking) induced in larval ORNs. While we cannot rule out bleaching or decline in ORN responses during longer (1 min) bouts of optogenetic stimulations, a recent electrophysiology analysis of larval ORNs to long odor exposures showed that ORNs continued to respond to (5 and 20 min) odor exposures, with no complete adaptation, and no change in their ability to encode temporal information (Grillet et al., 2016). While light activation of *CsChrimson* leads to ORN excitation, we cannot rule out the possibility of ORN inhibition that might occur upon stimulus withdrawal. Recent studies have shown that peripheral inhibition is comparable to excitation in encoding sensory signals and just as effective in eliciting behavioral responses in the animal (Cao et al., 2017). In the case of larvae expressing *CsChrimson* in ORN::42a, we noted behavior response to stimulus decrements both in the presence and absence of a directional cue, anisole. In the case of larvae expressing *CsChrimson* in ORN::7a, we observed behavior response to stimulus increments only in presence of the directional cue but not in its absence. More studies using different dilutions of anisole as well as different odorants as directional cues are needed for a clearer picture of the role of larval ORNs in driving behavioral responses to increasing and decreasing stimuli. Our optogenetic analyses were restricted to only seven ORNs, which constitute only 33% of all larval ORNs. Similarly, we probed the temporal diversity of ORNs using only three different frequencies of ORN stimulation. Despite a tight correlation between light-dependent *CsChrimson* activation and the physiological activity induced in ORNs expressing *CsChrimson* (Hernandez-Nunez et al., 2015), we acknowledge that any conclusions derived from our optogenetic experiments regarding ORN properties should be confirmed using natural odor stimuli.

### General Impact of the Study

Overall, the present study challenges traditional methods of incorporating ORN activity into computational models built to predict animal behavior. The ability of larval ORNs to encode different temporal patterns of activation as well as differentiate between stimulus increments and decrements may significantly increase the coding capacity of the olfactory circuit to identify and navigate toward an odor source. Knowledge of how ORN activity patterns and their weighted contributions influence odor coding may eventually reveal how peripheral information is organized and transmitted to subsequent layers of a neural circuit.

## Author Contributions

DC, DS, and DM designed the research. DC, SO, JA, MT, DK, and AM performed the experiments. DC, SO, JA, DS, and DM analyzed the data. DM wrote the article with the help of all other authors.

## Conflict of Interest Statement

The authors declare that the research was conducted in the absence of any commercial or financial relationships that could be construed as a potential conflict of interest.

## Supplementary Material

TABLE S1 Behavioral responses to test odorants. Mean ± SEM values for the four navigational parameters measured in the odorant pre-exposure experiment (Figure 1A) are provided.

TABLE S2 Behavioral responses to optogenetic stimulation. Mean ± SEM values for all navigational parameters measured (except run ratio) in the optogenetic experiment (Figures 3, 4) for control **(A)** and test lines **(B–H)** are provided.
