# Supplementary material for: Behavior Responses to Chemical and Optogenetic Stimuli in Drosophila Larvae
Source: Front Behav Neurosci. 2018 Dec 21;12:324. doi: 10.3389/fnbeh.2018.00324 (PMC6308144; doi:10.3389/fnbeh.2018.00324)
Supplement: TABLE S1 — Behavioral responses to test odorants. Mean ± SEM values for the four navigational parameters measured in the odorant pre-exposure experiment (Figure 1A) are provided. [file Table_1.pdf]

Supplementary Table S1

A Behavioral responses to test odorants (Mean ± SEM)

Odor	Curve Rating	Runs per Track	Run Speed (mm/s)	Run Length (mm)
**Paraffin oil**	2.62 ± 0.204	4.84 ± 0.163	0.275 ± 0.004	9.11 ± 0.320
**Trans-2-hexenal**	2.79 ± 0.262	5.53 ± 0.240	0.299 ± 0.007	8.57 ± 0.432
**6-methyl-5-hepten-2-ol**	3.20 ± 0.315	5.29 ± 0.273	0.313 ± 0.007	8.99 ± 0.478
**2-acetylpyradine**	2.35 ± 0.170	4.15 ± 0.236	0.353 ± 0.010	14.0 ± 0.809
**methyl phenyl sulfide**	2.42 ± 0.234	3.90 ± 0.251	0.375 ± 0.011	15.8 ± 0.964
**anisole**	2.10 ± 0.122	5.50 ± 0.298	0.308 ± 0.008	9.57 ± 0.578
**2,5-dimethylpyrazine**	2.60 ± 0.232	5.21 ± 0.325	0.343 ± 0.010	12.4 ± 0.854
**1-pentanol**	2.39 ± 0.178	4.21 ± 0.244	0.364 ± 0.011	13.9 ± 0.770
**4-hexen-3-one**	2.18 ± 0.167	5.11 ± 0.328	0.335 ± 0.010	12.4 ± 0.804
**acetal**	2.37 ± 0.155	3.93 ± 0.177	0.420 ± 0.009	15.9 ± 0.772
**2-nonanone**	2.46 ± 0.136	4.49 ± 0.274	0.320 ± 0.009	12.7 ± 0.691
**4-methyl-5-vinylthiazole**	3.74 ± 1.08	5.38 ± 0.281	0.312 ± 0.008	10.1 ± 0.577
**pentyl acetate**	2.47 ± 0.311	5.21 ± 0.279	0.323 ± 0.009	11.2 ± 0.643
**4,5-dimethythiazole**	2.32 ± 0.180	5.12 ± 0.276	0.368 ± 0.009	12.3 ± 0.689
**trans-3-hexen-1-ol**	2.79 ± 0.319	4.77 ± 0.278	0.313 ± 0.008	10.8 ± 0.621
**trans,trans-2,4-nonadienal**	2.38 ± 0.217	4.54 ± 0.272	0.316 ± 0.008	11.9 ± 0.649
**gernayl acetate**	2.63 ± 0.235	4.55 ± 0.271	0.309 ± 0.008	11.8 ± 0.638
**3-octanol**	2.53 ± 0.188	5.64 ± 0.268	0.286 ± 0.007	8.87 ± 0.495
**2-methoxyphenyl acetate**	2.89 ± 0.382	4.59 ± 0.275	0.336 ± 0.010	12.8 ± 0.781
