# Supplementary material for: Behavior Responses to Chemical and Optogenetic Stimuli in Drosophila Larvae
Source: Front Behav Neurosci. 2018 Dec 21;12:324. doi: 10.3389/fnbeh.2018.00324 (PMC6308144; doi:10.3389/fnbeh.2018.00324)
Supplement: TABLE S2 — Behavioral responses to optogenetic stimulation. Mean ± SEM values for all navigational parameters measured (except run ratio) in the optogenetic experiment (Figures 3, 4) for control (A) and test lines (B–H) are provided. [file Table_2.pdf]

## A. Behavioral responses to optogenetic stimulation (Mean ± SEM): Control

| Stimulation Frequency | Minute | Curve Rating | Response Index | Runs per Track | Curvature | Direction | Run Speed (Towards) | Run Length (Towards) | Run Speed (Away) | Run Length (Away) |
|---|---|---|---|---|---|---|---|---|---|---|
| 0.04 Hz | 1 | 1.212 ±0.03 | 0.089 ±0.053 | 1.709 ±0.149 | -0.598 ±0.958 | 85.19 ±3.333 | 0.417 ±0.028 | 20.264 ±2.108 | 0.347 ±0.031 | 15.967 ±2.093 |
| | 2 | 1.577 ±0.127 | 0.238 ±0.05 | 2.176 ±0.167 | -0.732 ±1.194 | 77.604 ±2.879 | 0.33 ±0.02 | 14.301 ±1.353 | 0.255 ±0.024 | 9.027 ±1.601 |
| | 3 | 1.246 ±0.052 | 0.129 ±0.056 | 1.921 ±0.139 | 1.261 ±0.961 | 86.066 ±3.415 | 0.322 ±0.024 | 15.695 ±1.629 | 0.26 ±0.025 | 10.839 ±1.695 |
| 1 Hz | 1 | 1.218 ±0.031 | 0.011 ±0.052 | 1.652 ±0.117 | 0.917 ±0.899 | 88.249 ±3.32 | 0.399 ±0.035 | 20.751 ±2.463 | 0.385 ±0.027 | 17.008 ±2.061 |
| | 2 | 1.319 ±0.057 | 0.168 ±0.05 | 2.679 ±0.236 | 2.271 ±1.347 | 81.255 ±2.667 | 0.253 ±0.021 | 11.08 ±1.363 | 0.2 ±0.023 | 6.544 ±1.237 |
| | 3 | 1.203 ±0.045 | 0.136 ±0.053 | 2.205 ±0.231 | -0.239 ±1.712 | 82.235 ±3.247 | 0.214 ±0.023 | 9.406 ±1.442 | 0.181 ±0.022 | 7.028 ±1.276 |
| Constant | 1 | 1.401 ±0.084 | 0.02 ±0.06 | 1.951 ±0.161 | 0.215 ±1.083 | 91.512 ±3.52 | 0.37 ±0.028 | 16.571 ±2.041 | 0.308 ±0.026 | 13.308 ±1.777 |
| | 2 | 1.352 ±0.077 | 0.111 ±0.063 | 2.824 ±0.219 | -0.147 ±1.161 | 85.297 ±3.056 | 0.244 ±0.019 | 9.496 ±1.258 | 0.236 ±0.019 | 7.817 ±1.283 |
| | 3 | 1.373 ±0.177 | 0.117 ±0.056 | 2.194 ±0.171 | 0.758 ±1.319 | 85.098 ±3.194 | 0.299 ±0.023 | 13.385 ±1.543 | 0.22 ±0.025 | 8.599 ±1.493 |

## B. Behavioral responses to optogenetic stimulation (Mean ± SEM): Or7a

| Stimulation Frequency | Minute | Curve Rating | Response Index | Runs per Track | Curvature | Direction | Run Speed (Towards) | Run Length (Towards) | Run Speed (Away) | Run Length (Away) |
|---|---|---|---|---|---|---|---|---|---|---|
| 0.04 Hz | 1 | 1.692 ±0.264 | -0.043 ±0.058 | 1.925 ±0.172 | 0.47 ±1.419 | 97.875 ±3.711 | 0.658 ±0.087 | 31.442 ±5.68 | 0.51 ±0.057 | 21.102 ±3.747 |
| | 2 | 1.554 ±0.09 | 0.105 ±0.063 | 3.056 ±0.256 | -2.604 ±1.428 | 90.191 ±3.32 | 0.404 ±0.034 | 14.232 ±2.105 | 0.352 ±0.027 | 8.79 ±1.593 |
| | 3 | 1.702 ±0.204 | 0.134 ±0.068 | 2.581 ±0.246 | -0.407 ±1.245 | 83.635 ±3.59 | 0.428 ±0.038 | 17.48 ±2.554 | 0.36 ±0.043 | 11.542 ±2.291 |
| 1 Hz | 1 | 1.372 ±0.086 | -0.062 ±0.063 | 1.82 ±0.157 | 1.93 ±1.026 | 89.279 ±4.041 | 0.57 ±0.059 | 24.513 ±3.981 | 0.781 ±0.055 | 34.779 ±3.864 |
| | 2 | 1.86 ±0.198 | 0.052 ±0.063 | 3.941 ±0.311 | -0.15 ±1.471 | 87.32 ±2.989 | 0.324 ±0.028 | 8.449 ±1.447 | 0.338 ±0.036 | 8.038 ±1.606 |
| | 3 | 1.307 ±0.055 | 0.079 ±0.069 | 2.237 ±0.224 | -3.034 ±1.561 | 88.101 ±3.899 | 0.413 ±0.059 | 19.937 ±3.49 | 0.313 ±0.035 | 10.296 ±1.989 |
| Constant | 1 | 1.39 ±0.071 | -0.028 ±0.061 | 2.058 ±0.161 | -0.799 ±1.236 | 93.072 ±3.669 | 0.586 ±0.061 | 23.638 ±3.736 | 0.564 ±0.044 | 20.458 ±2.54 |
| | 2 | 2.039 ±0.22 | 0.093 ±0.066 | 3.415 ±0.254 | 4.423 ±2.374 | 92.561 ±3.268 | 0.421 ±0.038 | 15.917 ±2.56 | 0.33 ±0.025 | 8.678 ±1.569 |
| | 3 | 1.491 ±0.12 | 0.024 ±0.07 | 2.679 ±0.26 | -1.32 ±1.696 | 88.214 ±3.68 | 0.369 ±0.05 | 17.791 ±3.027 | 0.427 ±0.056 | 18.98 ±3.54 |

## C. Behavioral responses to optogenetic stimulation (Mean ± SEM): Or42a

| Stimulation Frequency | Minute | Curve Rating | Response Index | Runs per Track | Curvature | Direction | Run Speed (Towards) | Run Length (Towards) | Run Speed (Away) | Run Length (Away) |
|---|---|---|---|---|---|---|---|---|---|---|
| 0.04 Hz | 1 | 1.527 ±0.155 | -0.073 ±0.056 | 2.655 ±0.211 | 1.338 ±1.163 | 1.338 ±1.163 | 0.384 ±0.04 | 13.458 ±2.333 | 0.418 ±0.037 | 14.065 ±2.098 |
| | 2 | 1.314 ±0.057 | 0.045 ±0.056 | 2.605 ±0.219 | 0.044 ±2.158 | 91.498 ±3.087 | 0.389 ±0.038 | 15.319 ±2.296 | 0.288 ±0.029 | 9.324 ±1.691 |
| | 3 | 1.825 ±0.149 | 0.149 ±0.065 | 4.207 ±0.378 | 2.016 ±3.034 | 88.267 ±2.978 | 0.28 ±0.021 | 7.157 ±1.003 | 0.23 ±0.022 | 4.671 ±0.95 |
| 1 Hz | 1 | 1.42 ±0.085 | 0.053 ±0.06 | 2.407 ±0.194 | 0.376 ±1.72 | 90.63 ±3.29 | 0.441 ±0.042 | 19.203 ±2.767 | 0.374 ±0.035 | 13.521 ±2.242 |
| | 2 | 2.044 ±0.386 | 0.226 ±0.054 | 2.778 ±0.302 | -3.082 ±2.28 | 75.319 ±3.057 | 0.345 ±0.031 | 13.893 ±1.886 | 0.275 ±0.031 | 7.489 ±1.709 |
| | 3 | 1.685 ±0.219 | 0.306 ±0.063 | 3.772 ±0.319 | 3.467 ±2.361 | 82.064 ±3.078 | 0.358 ±0.026 | 12.039 ±1.596 | 0.208 ±0.022 | 3.132 ±0.647 |
| Constant | 1 | 1.441 ±0.106 | 0.054 ±0.062 | 2.01 ±0.174 | -2.176 ±1.616 | 89.976 ±3.52 | 0.507 ±0.046 | 24.836 ±3.228 | 0.437 ±0.047 | 17.962 ±2.813 |
| | 2 | 1.418 ±0.12 | 0.106 ±0.065 | 2.206 ±0.183 | 1.404 ±1.776 | 86.387 ±3.46 | 0.441 ±0.04 | 21.358 ±2.424 | 0.417 ±0.042 | 16.289 ±2.716 |
| | 3 | 1.513 ±0.176 | 0.229 ±0.07 | 3.861 ±0.314 | -5.633 ±3.41 | 80.843 ±3.205 | 0.307 ±0.028 | 9.797 ±1.475 | 0.244 ±0.029 | 5.441 ±1.252 |

## D. Behavioral responses to optogenetic stimulation (Mean ± SEM): Or42b

| Stimulation Frequency | Minute | Curve Rating | Response Index | Runs per Track | Curvature | Direction | Run Speed (Towards) | Run Length (Towards) | Run Speed (Away) | Run Length (Away) |
|---|---|---|---|---|---|---|---|---|---|---|
| 0.04 Hz | 1 | 1.545 ±0.143 | 0.105 ±0.066 | 2.561 ±0.271 | 1.332 ±2.273 | 84.967 ±3.369 | 0.443 ±0.043 | 18.85 ±2.638 | 0.399 ±0.044 | 15.608 ±2.677 |
| | 2 | 1.94 ±0.537 | 0.111 ±0.066 | 2.587 ±0.258 | -1.23 ±3.008 | 85.394 ±3.576 | 0.332 ±0.032 | 11.269 ±1.853 | 0.321 ±0.036 | 10.482 ±2.152 |
| | 3 | 1.425 ±0.118 | 0.265 ±0.064 | 2.828 ±0.258 | 0.97 ±2.146 | 83.499 ±3.49 | 0.372 ±0.03 | 15.503 ±2.091 | 0.236 ±0.031 | 6.16 ±1.559 |
| 1 Hz | 1 | 1.376 ±0.064 | -0.025 ±0.062 | 3.019 ±0.253 | -3.657 ±3.056 | 91.88 ±2.907 | 0.369 ±0.032 | 13.901 ±1.98 | 0.4 ±0.035 | 14.712 ±2.054 |
| | 2 | 1.409 ±0.08 | 0.114 ±0.066 | 3.957 ±0.397 | 0.14 ±2.588 | 86.335 ±2.744 | 0.319 ±0.027 | 11.813 ±1.659 | 0.224 ±0.025 | 6.794 ±1.396 |
| | 3 | 1.759 ±0.452 | 0.111 ±0.074 | 2.625 ±0.299 | 2.622 ±2.983 | 90.631 ±3.781 | 0.38 ±0.032 | 14.518 ±2.195 | 0.241 ±0.027 | 7.269 ±1.351 |
| Constant | 1 | 1.486 ±0.103 | -0.049 ±0.049 | 2.896 ±0.204 | -0.073 ±1.709 | 91.378 ±2.696 | 0.338 ±0.029 | 10.995 ±1.756 | 0.347 ±0.024 | 10.646 ±1.523 |
| | 2 | 2.276 ±0.488 | 0.052 ±0.058 | 3.528 ±0.263 | 2.678 ±2.398 | 88.615 ±2.566 | 0.315 ±0.024 | 10.487 ±1.518 | 0.265 ±0.024 | 8.086 ±1.282 |
| | 3 | 1.391 ±0.093 | 0.19 ±0.067 | 2.791 ±0.22 | -1.317 ±2.101 | 81.588 ±3.336 | 0.363 ±0.032 | 13.862 ±1.834 | 0.311 ±0.034 | 10.856 ±2.049 |
