# Supplementary material for: Behavior Responses to Chemical and Optogenetic Stimuli in Drosophila Larvae
Source: Front Behav Neurosci. 2018 Dec 21;12:324. doi: 10.3389/fnbeh.2018.00324 (PMC6308144; doi:10.3389/fnbeh.2018.00324)
Supplement: Supplementary file 3 [file Table_3.pdf]

E. Behavioral responses to optogenetic stimulation (Mean + SEM): 45a

Stimulation Frequency	Minute	Curve Rating	Response Index	Runs per Track	Curvature	Direction	Run Speed (Towards)	Run Length (Towards)	Run Speed (Away)	Run Length (Away)
0.04 Hz	1	1.249 ±0.053	0.088 ±0.065	1.614 ±0.128	2.033 ±1.334	80.773 ±4.322	0.596 ±0.051	28.924 ±3.531	0.457 ±0.052	21.78 ±3.695
	2	1.346 ±0.067	0.19 ±0.062	2.471 ±0.271	2.013 ±1.54	76.71 ±3.442	0.373 ±0.036	14.85 ±2.378	0.364 ±0.055	13.117 ±3.467
	3	1.407 ±0.096	0.223 ±0.07	2.808 ±0.317	-0.602 ±1.658	89.645 ±3.573	0.434 ±0.043	21.103 ±2.685	0.251 ±0.035	7.696 ±1.914
1 Hz	1	1.299 ±0.048	-0.014 ±0.062	2.02 ±0.175	0.857 ±1.526	95.451 ±3.62	0.55 ±0.064	25.199 ±3.754	0.412 ±0.052	17.269 ±2.778
	2	1.371 ±0.065	-0.005 ±0.065	3.011 ±0.271	0.088 ±1.38	91.769 ±3.041	0.323 ±0.039	11.685 ±2.035	0.351 ±0.043	13.197 ±2.418
	3	1.445 ±0.093	-0.02 ±0.072	2.573 ±0.292	-2.896 ±1.432	91.665 ±4.038	0.354 ±0.037	12.445 ±2.291	0.381 ±0.041	12.988 ±2.329
Constant	1	1.605 ±0.187	-0.064 ±0.066	2.488 ±0.278	-0.994 ±1.548	91.478 ±3.528	0.343 ±0.049	13.314 ±2.997	0.408 ±0.056	16.478 ±3.282
	2	1.353 ±0.062	-0.136 ±0.07	2.671 ±0.285	0.64 ±1.566	93.113 ±3.672	0.303 ±0.047	11.98 ±3.123	0.305 ±0.037	12.018 ±2.369
	3	1.523 ±0.17	0.021 ±0.076	3.063 ±0.322	-0.883 ±1.767	93.6 ±3.959	0.294 ±0.037	8.107 ±1.812	0.276 ±0.029	7.982 ±1.614

F. Behavioral responses to optogenetic stimulation (Mean + SEM): 45b

Stimulation Frequency	Minute	Curve Rating	Response Index	Runs per Track	Curvature	Direction	Run Speed (Towards)	Run Length (Towards)	Run Speed (Away)	Run Length (Away)
0.04 Hz	1	1.298 ±0.052	-0.022 ±0.062	1.741 ±0.114	0.683 ±0.835	87.801 ±3.963	0.559 ±0.044	23.1 ±3.194	0.629 ±0.045	31.151 ±3.24
	2	1.509 ±0.097	0.04 ±0.063	2.253 ±0.198	0.124 ±1.474	85.542 ±3.823	0.412 ±0.039	16.222 ±2.561	0.346 ±0.035	13.944 ±2.32
	3	1.606 ±0.149	0.117 ±0.072	3.08 ±0.29	3.629 ±1.327	90.251 ±3.691	0.373 ±0.032	12.674 ±1.953	0.262 ±0.027	8.51 ±1.664
1 Hz	1	1.582 ±0.167	-0.092 ±0.057	2.263 ±0.224	1.247 ±1.121	96.169 ±3.515	0.432 ±0.058	17.861 ±3.712	0.458 ±0.038	17.864 ±2.577
	2	1.712 ±0.219	0.066 ±0.067	2.8 ±0.295	1.072 ±1.403	87.491 ±3.533	0.439 ±0.043	17.72 ±2.547	0.399 ±0.04	12.682 ±2.333
	3	1.712 ±0.152	0.102 ±0.068	2.182 ±0.209	1.888 ±1.401	84.187 ±4.059	0.394 ±0.05	16.989 ±3.02	0.368 ±0.05	15.537 ±3.37
Constant	1	1.57 ±0.143	-0.034 ±0.06	2.009 ±0.157	-1.026 ±1.067	91.201 ±3.706	0.58 ±0.049	24.594 ±3.35	0.546 ±0.044	22.806 ±2.897
	2	1.59 ±0.3	0.029 ±0.062	2.293 ±0.224	-1.097 ±1.438	86.696 ±3.576	0.466 ±0.046	19.564 ±3.06	0.379 ±0.05	16.433 ±3.081
	3	1.965 ±0.184	0.21 ±0.065	2.78 ±0.239	1.581 ±2.291	79.933 ±3.642	0.39 ±0.032	14.422 ±1.98	0.337 ±0.03	8.068 ±1.509

G. Behavioral responses to optogenetic stimulation (Mean + SEM): 47a

Stimulation Frequency	Minute	Curve Rating	Response Index	Runs per Track	Curvature	Direction	Run Speed (Towards)	Run Length (Towards)	Run Speed (Away)	Run Length (Away)
0.04 Hz	1	1.331 ±0.067	-0.09 ±0.06	1.844 ±0.153	-0.474 ±1.129	95.188 ±3.629	0.523 ±0.077	26.41 ±5.251	0.547 ±0.058	24.924 ±3.493
	2	1.305 ±0.054	0.037 ±0.073	2.757 ±0.292	2.356 ±1.663	94.385 ±3.661	0.48 ±0.052	21.102 ±3.71	0.314 ±0.039	11.894 ±2.444
	3	1.34 ±0.063	0.084 ±0.075	2.815 ±0.26	1.112 ±1.434	91.132 ±3.862	0.417 ±0.049	17.92 ±2.885	0.34 ±0.038	9.554 ±2.463
1 Hz	1	1.447 ±0.1	-0.054 ±0.06	1.868 ±0.133	0.839 ±0.962	97.014 ±3.951	0.633 ±0.052	29.377 ±3.832	0.476 ±0.042	19.411 ±2.471
	2	1.466 ±0.103	0.059 ±0.07	2.654 ±0.292	-1.005 ±1.554	86.324 ±3.996	0.388 ±0.043	15.766 ±2.67	0.327 ±0.035	11.747 ±2.086
	3	1.8 ±0.305	0.159 ±0.072	2.743 ±0.397	1.397 ±1.79	87.695 ±3.89	0.34 ±0.039	15.204 ±2.699	0.298 ±0.041	8.154 ±1.989
Constant	1	1.367 ±0.078	0.021 ±0.075	1.733 ±0.147	-3.301 ±1.032	87.764 ±4.706	0.681 ±0.056	35.806 ±3.679	0.719 ±0.059	32.528 ±3.729
	2	1.592 ±0.11	0.089 ±0.072	2.538 ±0.255	-1.967 ±1.563	87.568 ±3.98	0.449 ±0.037	16.649 ±2.472	0.389 ±0.042	102.772 ±21.199
	3	1.635 ±0.18	0.103 ±0.073	2.848 ±0.279	1.409 ±1.434	89.398 ±4.029	0.393 ±0.051	15.766 ±3.078	0.324 ±0.043	10.822 ±2.616

H. Behavioral responses to optogenetic stimulation (Mean + SEM): 67b

Stimulation Frequency	Minute	Curve Rating	Response Index	Runs per Track	Curvature	Direction	Run Speed (Towards)	Run Length (Towards)	Run Speed (Away)	Run Length (Away)
0.04 Hz	1	1.327 ±0.057	-0.098 ±0.057	1.88 ±0.15	1.269 ±2.254	96.892 ±3.638	0.532 ±0.047	22.849 ±3.297	0.545 ±0.038	22.857 ±2.823
	2	1.575 ±0.114	0.004 ±0.063	2.714 ±0.264	-0.254 ±1.41	90.583 ±3.173	0.434 ±0.04	15.377 ±2.45	0.39 ±0.036	14.854 ±2.435
	3	1.343 ±0.076	0.153 ±0.065	2.416 ±0.253	0.376 ±1.29	83.491 ±3.705	0.45 ±0.048	21.227 ±3.419	0.253 ±0.029	7.97 ±2.016
1 Hz	1	1.513 ±0.083	-0.199 ±0.054	2.148 ±0.145	-0.059 ±1.004	104.378 ±3.354	0.545 ±0.067	21.204 ±4.007	0.517 ±0.033	21.006 ±2.186
	2	1.684 ±0.178	-0.03 ±0.061	2.936 ±0.227	0.868 ±1.187	95.227 ±3.217	0.492 ±0.047	17.883 ±2.801	0.34 ±0.03	10.614 ±1.71
	3	1.47 ±0.099	0.099 ±0.063	2.291 ±0.237	-2.218 ±1.484	86.634 ±3.867	0.488 ±0.051	20.471 ±3.147	0.356 ±0.042	11.029 ±2.26
Constant	1	1.381 ±0.055	0.066 ±0.053	1.982 ±0.131	-1.072 ±1.061	88.271 ±3.457	0.534 ±0.053	19.772 ±3.628	0.511 ±0.063	22.254 ±3.975
	2	1.439 ±0.101	0.045 ±0.064	2.441 ±0.224	-1.285 ±1.214	91.11 ±3.492	0.514 ±0.044	21.522 ±3.038	0.355 ±0.039	13.825 ±2.427
	3	1.615 ±0.156	0.062 ±0.068	2.597 ±0.212	0.867 ±1.557	87.741 ±3.863	0.42 ±0.051	17.526 ±2.981	0.388 ±0.044	14.885 ±2.908
